# Laryngeal assessment in reumatic disease patients

**DOI:** 10.1016/S1808-8694(15)31206-4

**Published:** 2015-10-20

**Authors:** Hugo Valter Lisboa Ramos, Jackeline Pillon, Eduardo M Kosugi, Reginaldo Fujita, Paulo Pontes

**Affiliations:** 1 Master in Otorhinolaryngology, Post-graduation, Department of Otorhinolaryngology and Head and Neck Surgery, UNIFESP - EPM.; 2 Speech Therapist, Department of Otorhinolaryngology and Head and Neck Surgery, UNIFESP - EPM.; 3 Otorhinolaryngologist, Post-graduation under course, Department of Otorhinolaryngology and Head and Neck, UNIFESP - EPM.; 4 Ph.D. in Medicine, Head of Clinical Pediatric Otorhinolaryngology, Discipline of Otorhinolaryngology and Head and Neck Surgery, UNIFESP - EPM.; 5 Full Professor, Faculty Professor, Department of Otorhinolaryngology and Head and Neck Surgery, UNIFESP - EPM. Department of Otorhinolaryngology and Head and Neck Surgery, UNIFESP - EPM.

**Keywords:** larynx, systemic lupus erythematous, systemic sclerosis, mixed connective tissue disease, bambu node

## Abstract

Rheumatic diseases usually promote several systemic disorders, which can affect blood vessels, mucosa and serosa of the aerodigestive tract. Scarce laryngeal involvement has been described in these patients and this study aims at investigating laryngeal alterations found in patients with rheumatic diseases. **Study design:** transversal cohort. **Material and method**: A transversal study was developed with systemic lupus erythematous, systemic sclerosis and mixed connective tissue disease’s patients. They were evaluated by means of clinical examinations and videolaryngoestroboscopy. **Results**: Twenty-seven patients were included in the study, 26 succeeded in completing the videolaryngoestroboscopy. Laryngeal abnormalities were seen in 11 of 12 patients with lupus, in all 11 patients with sclerodermia and in 3 patients with mixed connective tissue disease. Vocal fold bamboo node was observed in 5 patients and 92.3% of all patients presented laryngeal signs of gastroesophageal reflux disease. **Conclusion**: We noticed 5 vocal fold bamboo nodes and gastroesophageal reflux disease in almost all patients.

## INTRODUCTION

Rheumatic diseases comprise a heterogeneous group of entities producing connective tissue systemic disorders. Therefore, they may involve blood vessels and the aerodigestive serous and mucous structures, particularly the larynx.

The first laryngeal involvement reported in a systemic lupus erythematosus patient (SLE) was published in 1959 by Scarpelli et al. ^1^ and, since then, other cases have been sporadically described. In 1976, Smith et al. ^2^ reported ulcerations, stenosis and edema of cricoarytenoid units of two SLE patients. However, Tietel et al. ^3^ published a 97-case revision on laryngeal involvement in SLE patients, in which laryngeal edema was found in 28% of patients and vocal fold paralysis in 11%.

However, laryngeal involvement is not exclusive of SLE. In a study with 45 patients with fairly severe rheumatoid arthritis (RA) submitted to indirect laryngoscopy, Lawry et al. ^4^ detected abnormalities in 52% of cases. Diseases such as scleroderma and Sjögren’s syndrome have also presented laryngeal involvement. Murano et al. ^5^ described two cases of what is referred to as “bamboo nodules” and, after specific exams, those patients were diagnosed for autoimmune disease (Sjögren’s syndrome and systemic lupus erythematosus).

This study aims at identifying and describing laryngeal disorders in patients with SLE, scleroderma and mixed connective tissue disease (MCTD) by means of videostroboscopy exam of the larynx.

## METHOD

This is a transversal study carried out at the Department of Otorhinolaryngology and Head and Neck Surgery, *Federal University of Sao Paulo / Escola Paulista de Medicina (UNIFESP/EPM)*.

The study comprised 12 patients with SLE, 11 with scleroderma and 3 with mixed connective tissue disease (MCTD), totalizing 27 patients. All patients were women within the age range of 19 and 63 years, mean age of 41.2. Relative to race, 13 were Mulatto, 7 were Black and 6 were Caucasian.

Patients were included in the study as volunteers, regardless of presence of laryngeal disorder and duration of disease. One patient with MCTD and 2 with scleroderma presented associated Sjögren’s syndrome. All were laboratory and clinically diagnosed as having rheumatic disease. Unconfirmed cases of rheumatic diseases were excluded from the study. Seventeen individuals presented positive autoantibodies: antinuclear antibody (ANA) found in 16 patients (59.2%) and anti-RNP in 11 patients (40.7%). Fifteen (57.7%) patients were using prednisone daily at different doses (Tables 1, 2 and 3).

**Table 1:** Patients with SLE included in the study.

Patient	Age	· Findings	· Antibodies	Medication	
1		46	· Signs of GERDa	· ANAb	Prednisone
	· Bamboo nodules	· Anti-SMd	Chloroquine		
		· Anti-ROe	Methotrexate		
2	48	· Signs of GERDa		Prednisone	
3	48	· Signs of GERDa	· ANAb NSAID	Thalidomide	
4	45	· Signs of GERDa	Chloroquine	Prednisone	
5	47	· Anti-RNPc	· ANAb	Prednisone	
6	35	· Signs of GERDa	· ANAb	Prednisone	
	· Bamboo nodules	· Anti-RO/LAe	Azathioprine		
7	32	· Signs of GERDa	· ANAb	Prednisone	
			Chloroquine		
			Methotrexate		
			Azathioprine		
8	49	· Signs of GERDa	· ANAb		
		· Anti-RNPc			
9	45	· Signs of GERDa		Prednisone	
10	56	· Signs of GERDa	· ANAb	Prednisone	
		· Anti-SMd	Azathioprine		
		· Anti-RNPc			
11	28	· Signs of GERDa	· Anti-RNPc	Prednisone	
	· Bamboo nodule	· Anti-ROe	Chloroquine		
12	34	· Signs of GERDa	· ANAb	Prednisone	
		· Anti-RNPc	Chloroquine		
		· FRf			

– gastroesophageal reflux disease

– antinuclear antibody

– anti-ribonucleoprotein antibody

– anti-antigen SM antibody

– anti-antigen RO or LA antibody

– rheumatoid factor

**Table 2:** Patients with Scleroderma included in the study.

Patient	Age	Findings	Antibodies	Medication
1	44	· Signs of GERDa	· ANAb	Prednisone
		· Anti-RNPd	Methotrexate	
2	44	· Signs of GERDa		
		· Vascular dilations		
3	22	· Signs of GERDa	· ANAb	
4	63	· Signs of GERDa		Lidocaine IV
5	54	· Signs of GERDa		Prednisone for 10 years
		· Laryngeal hypertrophy		
6	63	· Signs of GERDa	· ANAb	
			· Anti-RNPc	
			· Anti-ROd	
7	32	· Signs of GERDa		
8	48	· Signs of GERDa	· ANAb	
		· Bilateral vascular dilations		
9	22	· Signs of GERDa		D-Penicillamine
		· Chink + Vocal nodule		
10	19	· Signs of GERDa		
11	29	· Signs of GERDa	· ANAb	
			· Anti-RNPc	
			· Anti-ROd	
			· FR^e^	

– gastroesophageal reflux disease

– antinuclear antibody

– anti-ribonucleoprotein antibody

– anti-antigen SM antibody

– rheumatoid factor

e – anti-antigen RO or LA antibody

**Table 3:** Patients with MCTD included in the study.

Patient	Age	Findings	Antibodies	Medications
1	51	· Signs of GERDa		Prednisone
	· Chink			
2	31	· Signs of GERDa		Chloroquine
		· Bamboo nodule		
3	36	· Bamboo nodule		Prednisone Chloroquine

– gastroesophageal reflux disease

Patients were submitted to otorhinolaryngological clinical examination and videolaryngostroboscopy using a 70° rigid telescope introduced into the oral cavity under topic lidocaine anesthetic spray.

## RESULTS

Out of 27 patients formerly included in the study, 26 could undergo videolaryngostroboscopy. One patient with scleroderma was excluded as she did not succeed in completing assessment due to intense nausea reflex.

**Figure 1:**
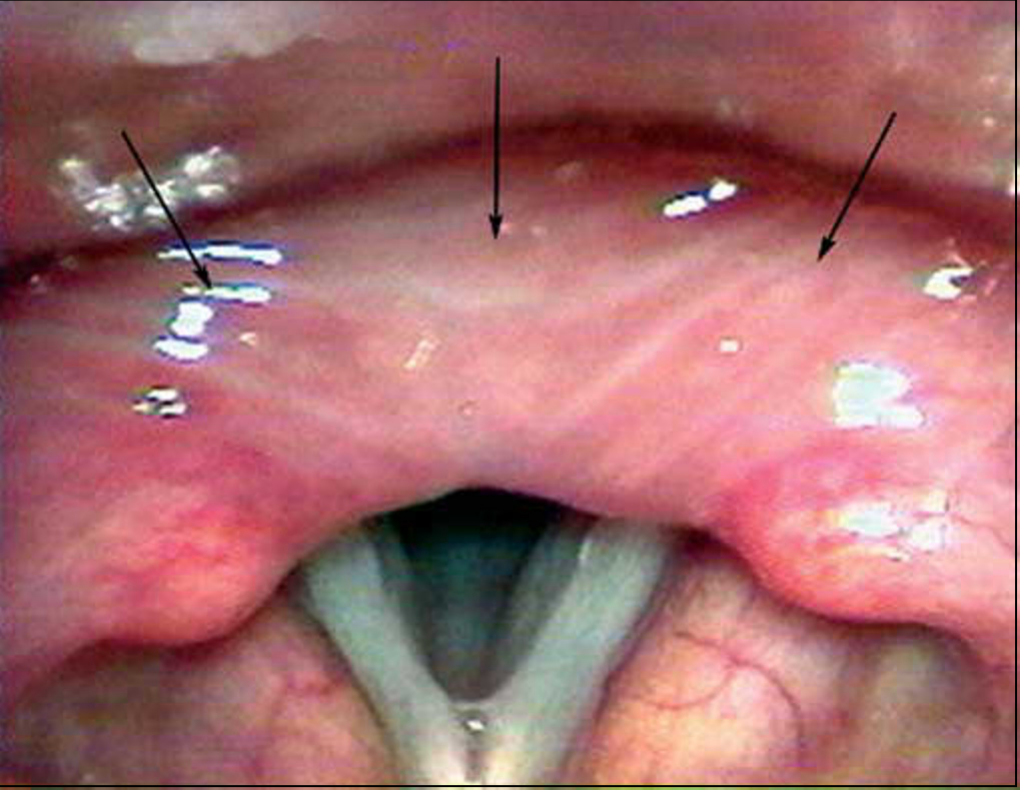
Edema of the retrocricoid region (Arrows).

Among 12 patients with SLE, 11 (91.6%) presented some type of laryngeal disorder (Table 1). The most frequent alteration found in 11 patients was the presence of edema and thickening of the interarytenoid region, which has been described as a sign of gastroesophageal reflux disease (GERD) (Figure 1). Among these patients, 9 presented clinical complaints of globus pharyngeus, hoarseness and dry cough.

**Figure 2:**
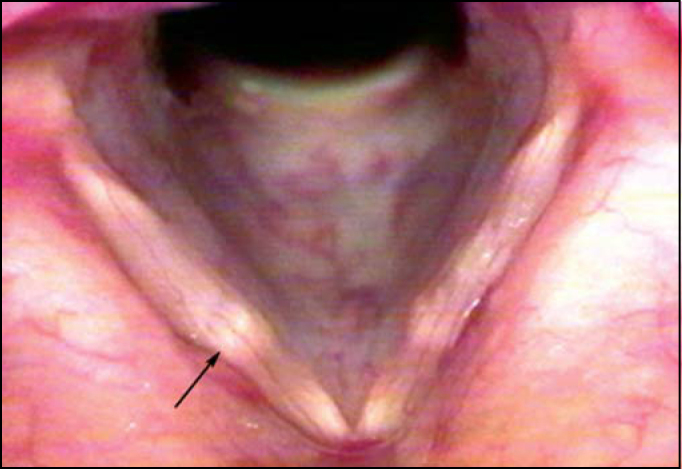
Bamboo nodule on the middle third of the right vocal fold (Arrow).

**Figure 3:**
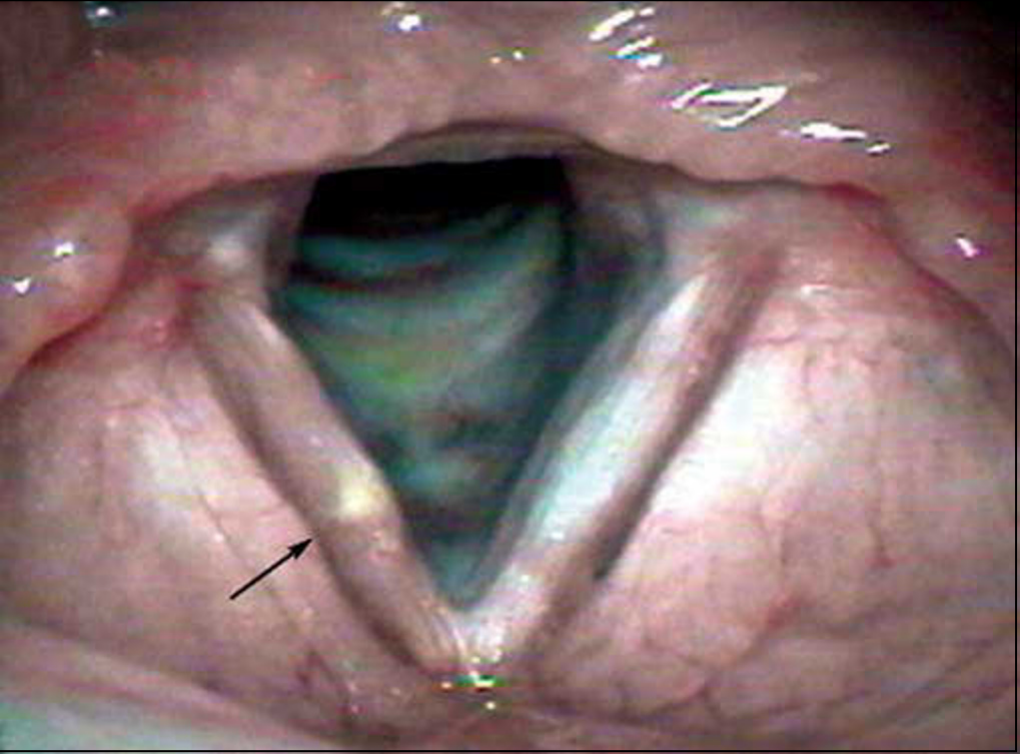
Bamboo nodule on the middle third of the right vocal fold (Arrow).

Other 3 patients showed yellowish and transversal deposit lesions of *lamina propria*, classically referred as “bamboo nodules” (Figures 2 and 3). A small unilateral vocal sulcus was observed in another patient.

All patients (11) with scleroderma presented laryngeal alterations (Table 2). Alterations suggestive of GERD were observed in 11 patients, out of which 6 had clinical complaints. Two patients presented blood vessels dilation of vocal folds and 2 had vocal nodules. Signs of laryngeal hypertrophy were observed in 1 patient (vocal fold hypertrophy on the prominence of vocal processes).

Out of 3 patients with MCTD, 2 presented “bamboo nodules” and 2 showed alterations suggestive of GERD (Table 3). Ten out of 26 patients presented hoarseness and 5 complained of dysphagia.

## DISCUSSION

Laryngeal alterations in patients with rheumatic diseases have been described and may be present in different diseases and in varied forms. Tietel et al. ^3^, based on a 97-case revision on SLE with laryngeal involvement, classified this involvement into 9 categories: mucous inflammation, infection, vasculitis, paralysis of the vocal fold, cricoarytenoid arthritis, subglottic stenosis, inflammatory tumor, rheumatoid nodules and epiglottis. The authors support the fact that these alterations may be a sign of imminent exacerbation of disease similarly to other indicators of inflammatory activity.

In our study, out of 12 patients with SLE, 3 exhibited rheumatoid vocal nodules, which are disorders found in the list of 9 categories previously described. Although scarce cases of rheumatoid nodules have been described some decades ago, Hosako-Naito et al. ^6^ were the most dedicated group to study these lesions. They described them in patients with SLE, scleroderma and Hashimoto’s thyroiditis or “bamboo nodules”.

In a literature revision, Murano et al. ^5^ found 6 described cases of patients with SLE, scleroderma, Sjögren’s syndrome and MCTD. The authors reported two cases of suspected autoimmune disease with laryngological findings of “bamboo nodules” and support nomenclature standardization for bamboo vocal nodules. Histologically, this lesion shows areas of linear granulomas with central necrosis surrounded by macrophages^5^. However, the laryngoscopic view of lesion is very similar to an intracordal cyst, even when examined by an expert professional.

We identified 1 patient with bowed vocal folds and arytenoid cartilages presenting prominent vocal processes. Regardless of the typical aspects of presbylarynx in these findings, we believe that they are caused by rheumatic disease, considering that the patient was 54 years of age. Wolman et al. ^7^ performed a *post-mortem* study in 8 patients with rheumatoid arthritis and verified that they all had histological signs of neurogenic muscle atrophy. In addition, 4 patients showed signs of chronic myositis associated with nerve degeneration.

So far, our study seems to be unique in assessment of patients with SLE, scleroderma and MCTD, regardless of presence of laryngeal complaint and of dysphonic process. Out of 26 patients, alterations were verified in 8 cases, which, we believe, are laryngeal manifestations of rheumatic diseases: 5 lesions suggestive of bamboo vocal nodules, 2 vocal folds with blood vessels dilatation, and 1 patient with hypertrophy of the thyroarytenoid muscle. Even though this type of disorder has not been described by other authors, it is interesting to notice it causes great discomfort to the patient and may contribute to worsen laryngeal affections.

Moreover, we observed signs of GERD in most patients (92.3%), as well as hoarseness, dry cough and feeling of a foreign body in the throat in 70.8% of them. Perhaps esophageal involvement by rheumatic disease, associated with frequent use of medicines that predispose to GERD, is responsible for the increased number of patients with GERD signs.

## CONCLUSION

In this study, we could observe laryngeal lesions related to rheumatic diseases in 8 of 26 patients assessed. We also identified 5 lesions suggestive of “bamboo nodules”, which represent the greatest number ever described. In addition, we observed that all patients showed laryngeal signs of GERD.

## References

[1] Scarpelli DG, McCoy FW, Scott JK (1959). Acute lupus erythematosus with laryngeal involvement. N Engl J Med.

[2] Smith GA, Ward PH, Berci G (1978). Laringeal lupus erythematosus. J Laryngol Otol.

[3] Teitel AD, Mackenzie CR, Stern R, Paget SA (1992). Laryngeal involvement in lupus erythematosus. Semin Arthritis Rheum.

[4] Lawry GV, Finerman ML, Hanafee WN, Mancuso AA, Fan PT, Bluestone R (1994). Laryngeal involvement in rheumatoid arthritis. Arthritis and Rheumatism.

[5] Murano E, Hosako-Naito Y, Tayama N, Oka T, Miyaji M, Kumada M, Niimi S (2001). Bamboo node: Primary vocal fold lesion as evidence of auto-imuneimune disease. Journal of Voice.

[6] Hosako-Naito Y (1999). Diagnosis and physiopathology of laryngeal deposits in auto-imuneimmune disease. ORL.

[7] Wolman L, Darke CS (1965). Young. The larynx in rheumatoid arthritis. J Laryngol Otol.

